# The airbag problem–a potential culprit for bench-to-bedside translational efforts: relevance for Alzheimer’s disease

**DOI:** 10.1186/2051-5960-1-62

**Published:** 2013-09-23

**Authors:** Dimitrije Krstic, Irene Knuesel

**Affiliations:** 1 Institute of Pharmacology and Toxicology, University of Zurich, Zurich, CH-8057, Switzerland

**Keywords:** Alzheimer’s disease AD, Amyloid Beta Aβ, Amyloid precursor protein APP, Reelin, Neuroinflammation, Neurodegeneration, Oxidative stress, Synaptic plasticity, Acute-phase reaction

## Abstract

For the last 20 years, the “amyloid cascade hypothesis” has dominated research aimed at understanding, preventing, and curing Alzheimer’s disease (AD). During that time researchers have acquired an enormous amount of data and have been successful, more than 300 times, in curing the disease in animal model systems by treatments aimed at clearing amyloid deposits. However, to date similar strategies have not been successful in human AD patients. Hence, before rushing into further clinical trials with compounds that aim at lowering amyloid-beta (Aβ) levels in increasingly younger people, it would be of highest priority to re-assess the initial assumption that accumulation of Aβ in the brain is the primary pathological event driving AD. Here we question this assumption by highlighting experimental evidence in support of the alternative hypothesis suggesting that APP and Aβ are part of a neuronal stress/injury system, which is up-regulated to counteract inflammation/oxidative stress-associated neurodegeneration that could be triggered by a brain injury, chronic infections, or a systemic disease. In AD, this protective program may be overridden by genetic and other risk factors, or its maintenance may become dysregulated during aging. Here, we provide a hypothetical example of a hypothesis-driven correlation between car accidents and airbag release in analogy to the evolution of the amyloid focus and as a way to offer a potential explanation for the failure of the AD field to translate the success of amyloid-related therapeutic strategies in experimental models to the clinic.

## Introduction

At the time of its wording in 1991/92, the “amyloid cascade hypothesis” [1] provided a logical explanation for the distinct amyloid-beta (Aβ) plaque pathology in patients diagnosed with Alzheimer’s Disease (AD). This hypothesis was supported by the direct link between AD and dominant mutations in either the *amyloid precursor protein* (APP) or enzymes that are involved in the production of Aβ peptides, such as *presenilin* 1 or 2 (PS1/2) [2-5]. Subsequently, numerous *in vitro* studies supported a toxic effect of Aβ peptides and by that “approved” its causative role in the disease etiology. However, mice that selectively overproduce Aβ peptides (BRI2-Aβ_1-42_) and develop plaque pathology display no signs of progressive cognitive impairments or neurodegeneration [6,7]. In addition, knock-in strategies to induce genetic mutations in APP or PS1 in rodents have been proven not to be sufficient to evoke AD-like phenotypes [8,9]. The development of certain features of the disease in animal models depends on transgene overexpression [10,11] and combinations of mutations [12-14]. Only very strong promoters and the presence of multiple mutations in mice, a combination never occuring in AD patients, triggers most of the neuropathological and behavioral phenotypes observed in humans, albeit in very young animals [15]. Nevertheless, transgenic-AD animals became state-of-the art tools in the community, and Aβ remained the proposed principle toxic and causative agent of the disease; first as monomer, then as an insoluble aggregate, followed by a soluble oligomer, and now a combination of all of these forms [16-18].

## Review

### Aβ–an acute-phase peptide

However, despite its proposed toxic/causative role in AD, physiological/protective roles for Aβ peptides have been described [19-21]. In addition to these neurotrophic effects of Aβ and its neurogenic effect on adult neural stem cells [22], Aβ peptides have been shown to stimulate synaptic function at physiological, picomolar, levels [23]. Increases in synaptic activity, through NMDA receptor activation, induce Aβ production [24], which at high, nanomolar, concentration potently depresses synaptic activity [23]. Hence, Aβ might serve as a suitable synaptic guardian against excitotoxicity. Furthermore, Aβ peptides capture metal ions such as Zn, Fe, and Cu, potentially reducing oxidative stress to neurons [25]. This is consistent with the notion that increased oxidative damage is an early feature in AD development [26-29] and with findings that subsequent increases in Aβ accumulation correlates with the decrease in oxidative stress [27,30]. Similarly, physiological levels of Aβ peptides in plasma and cerebrospinal fluid (CSF) are reported to protect circulating lipoproteins from oxidation [31].

It has been also demonstrated that Aβ possesses anti-inflammatory [32] and anti-microbial functions [33]. This potential immune function of Aβ is in line with studies showing induction of APP and Aβ production in brains of non-transgenic mice infected with *C. pneumonia*[34] or *Herpes simplex virus type 1*[35]. One should also keep in mind that although APP production in the brain is most abundant in neurons, astrocytes also produce APP and Aβ and this production is elevated by pro-inflammatory cytokines [36]. Interestingly, as has been noted in the brain, stimulation of the peripheral immune system induces expression of APP/Aβ in both T-lymphocytes [37] and CD14-positive mononuclear phagocytes [38]. Moreover, in humans correlations between neurospirochetosis and AD [39], and between periodontal infections and AD [40] are well established. In addition, young individuals exposed to elevated air pollution levels show neuroinflammatory responses with a significant induction of Aβ_1-42_ at autopsy [41].

Hence, in the context of Alzheimer’s disease progression, Aβ accumulation and plaque formation at sites of axonal swellings [42] and leakages [43,44] might be interpreted as the brain’s strategy to constrain such inflammation/oxidative stress-inducing hot-spots. This hypothesis is well in line with the observation that Aβ plaques form over 24 hours [45], possibly as an acute reaction to a burst or leakage of an axon as suggested in [44]. In parallel, increases in Aβ or other proteolytic fragments of APP at synaptic sites may act to protect vulnerable neurons from glutamate-mediated excitotoxicity [46].

### APP–a guardian of axonal and synapse integrity

Important, away from the spot-light of Aβ-driven research, endogenous functions of APP are also starting to emerge [47]. Of specific interest are observations in APP knock-out mice, which show relatively subtle phenotypes [48] but high vulnerability to mechanical insults and low neuroregenerative capacity [49]. The lack of APP expression in these animals is associated with enhanced susceptibility to kainic acid-induced epilepsy [50] and elevated mortality following cerebral ischemia [51]. These findings are consistent with the idea that the observed elevation of APP expression following traumatic brain injury [52-55], entorhinal cortex lesion [56], induced ischemia [57-59], systemic infection with a bacterial or a viral mimic [60,61], or administration of pro-inflammatory cytokine interleukin 1 [62,63] is a protective neuronal response to stress/injury, rather than a pathological event leading to an overproduction of Aβ. This is also well in line with the notion that while Aβ_1-42_ levels increase, APP levels decrease with advancing AD pathology [64]. In further support of this idea, head injury in *Drosophila* induces an increase in expression of *Drosophila* APP-Like Protein (dAPLP), and mutant flies lacking dAPLP have significantly higher mortality rates after injury than wild-type flies [65], suggesting an evolutionarily conserved role of APP in response to injury/stress. In rodents, double knock-out of APP and APLP-1 induces severe structural and functional synaptic deficits [66], pointing to the crucial role of APP family members in synapse development and maintenance.

Interestingly, a recently discovered “protective” mutation in APP (APP_A673T_) [67] results not only a delay of the onset of AD, but also in better cognitive performance of AD patients that carry the mutation. Important, this mutation leads not only to decreases in production of Aβ_x-42_, but also to a slight increase in levels of secreted APP ectodomain alpha (sAPPα) [67]. This is especially interesting as APP expression in aged animals is crucial for induction of long-term potentiation (LTP) and proper cognitive performance [68] and sAPPα was shown to reverse the deficits observed in APP knock-out mice [69].

In line with the hypothesis that impairments in axonal integrity and axonal transport represent a key contributing factor in the development of AD [44,70], APP has been shown to play a role in promoting kinesin-mediated fast axonal transport [71]. In addition, both a lack of APP [72] and an increase in proteolytic processing by γ-secretase [73] result in Aβ-independent [74] transport deficits. Together these findings suggest that stressful or injurious conditions in which APP endogenous functions are compromised, e.g. by a mutation in APP or its secretases, axonal transport deficits and subsequent synaptic loss may ensue. This view is supported by positron emission tomography (PET) imaging in human subjects in whom white matter atrophy and synaptic disconnection were observed early in AD pathogenesis [75-78]. Similarly, early white matter atrophy is also observed in women carrying the Apolipoprotein E (ApoE) allele ϵ4 [79], the major genetic risk factor for both familial and sporadic form of AD [80]. This is in agreement with the proposed endogenous function of ApoE in neuronal response to stress and injury [81,82].

### Non-demented individuals with AD neuropathology burden

Interestingly, approximately 25% of cognitively healthy elderly people have Aβ plaque and tangle pathology [83], sufficient to meet National Institute on Aging (NIA)-Reagan criteria for AD [84-86]. According to the amyloid cascade hypothesis these individuals would have developed dementia if they had lived longer, hence the name “prodromal Alzheimer patients”. Nevertheless, longitudinal cognitive tests in non-demented individuals above 90 years of age, but with high AD neuropathology, showed no evidence of cognitive decline three years before death [87]. Interestingly, while there was extensive overlap in Aβ peptides profile in AD patients and non-demented high pathology individuals [88], the levels of APP and Aβ_42_ were higher in the non-demented group [89]. In agreement with an anti-inflammatory role of Aβ peptides [32] there was less neuroinflammation in high-pathology non-demented individuals [90-92]. In addition, when compared to AD patients, these high-pathology non-demented individuals showed higher levels of neuroprotective ApoE and S100B, as well as angiogenic vascular endothelial growth factor (VEGF) [89]. Such non-demented individuals may, therefore, be successful in maintaining or recruiting additional protective pathways to fight age-associated neuroinflammatory stress [93] and its associated neurodegeneration [44]. If so, APP and Aβ may play a significant role in this protection. This idea is in line with a transcriptional profiling study, showing that a decisive point in AD neuropathogenesis is the induction of stress genes as a reaction of the brain to age-associated changes in lipid metabolism and increasing inflammatory stress [94].

While targeting inflammatory processes, as suggested by some studies and epidemiological data [95,96], may prove efficacious in preventing or delaying the development of AD, these treatments failed to show a beneficial effect in patients with symptomatic AD [97]. Hence, in order to design therapeutics that would have efficacy in already diagnosed, late-stage, AD patients we will have to exploit as yet undiscovered factors or pathways employed by the non-demented individuals, who exhibit high levels of neuropatholgical change. In a search for such protective factors, a recent genome-wide association study identified three of the top ten hits as single-nucleotide polymorphisms (SNPs) in the promoter region of a gene encoding for the glycoprotein Reelin [98]. Interestingly, Reelin is an extracellular signaling molecule shown to (i) modulate LTP, (ii) be a strong suppressor of Tau hyperphosphorylation, and (iii) modulate APP processing (reviewed in [99]). Important, compared to age-matched controls, Reelin levels are significantly lower in AD patients already at early stages of the disease [100,101], but in high-pathology non-demented individuals Reelin production is increased [98]. It was also demonstrated that employing histone deacetylase 2 (HDAC) inhibitor following severe experimentally-induced neurodegeneration virtually restored brain functionality [102]. This is specifically interesting since Reelin promoter is known to be sensitive to epigenetic changes [103]. Therefore, exploiting this and similar pathways might be a promising avenue for development of treatments for symptomatic AD patients. However, we are afraid that the pursuit of strategies other than those directly targeting Aβ may have been hindered by the early and prolonged adherence to the Amyloid theory.

### The airbag problem–a hypothetical example

The idea that the observed increase in Aβ levels as well as Aβ-containing senile plaques in AD patients is an adaptive response of the brain to an underlying stress/injury [44,94,104] has been put forward by a number of scientists [105-108]. Yet, many researchers continue to treat Aβ as the principle etiological agent in AD. While some might argue that an Aβ protective versus toxic role depends on the local concentration, it’s aggregation or oligomerization status, or the context in which Aβ is produced, we propose here an alternative possibility–namely that the scientific community is being misled by a seemingly overwhelming amount of literature that supports the Aβ hypothesis.

The rationale for making this strong and provocative statement against the amyloid hypothesis is easy to understand if you imagine a scenario in which someone, not knowing how a modern car works, is interpreting the significant association of car accidents with the presence of released airbags. Based on observations that released airbags are consistently observed in crashed cars, but never in intact cars, the airbag became the suspect and possible initiating cause of the accidents. Reinforced by the discovery that in a small percentage of car accidents (<1%) the spontaneous airbag release, due to a manufacturing defect, actually caused the accidence, the engineers design remote-controlled airbags to test the effect of their release in moving cars. Not knowing the real purpose of this protective device, the outcome and interpretation of this experiment–all performed with state-of-the-art technology, well-developed and executed strategies, and proper statistics–is absolutely clear: the airbag must be the cause. This strong evidence would certainly attract many specialists from different fields to investigate the airbag release mechanism in detail. They will eventually succeed, perhaps after years of hard work and accumulation of an overwhelming number of scientific papers, by changing only one important part of the release mechanism or installation of an airbag deployment preventer to hinder the experimentally induced airbag-associated car accidents. Since such intervention would prevent even small percentage of accidents caused by manufacturing defects that lead to unprovoked airbag deployment, the “airbag hypothesis” would become a well-established theory. Nevertheless the production of cars without airbags or removal of the airbags from all existing cars would not decrease the number of accidents in general-an obvious result if the real purpose of the airbag was known.

If we translate this hypothetical scenario to AD research, it might provide the explanation for the lack of translational success despite tremendous work and money invested in the past 20 years^a^. We argue here that the overwhelming data produced through the use of transgenic overexpression models of AD, which are not suitable for the understanding of the disease etiology, led the scientific community down the garden path. We would like to stress here, however, that the aim of this alternative view is not to put down the hard work being done by the AD-research community, but to encourage “outside the box” thinking toward initiating an important discussion and designing of future research directions–beyond Aβ. Finally, although we have focused in this article only on APP and Aβ in the context of Alzheimer’s disease, the implication of a potential “airbag problem” for bench-to-bedside translational efforts is not restricted to research on this protein and its derivatives nor to AD-research only.

## Conclusions

Alzheimer’s disease (AD) represents one of the major health-issues of modern society with no promising treatment on the horizon. A potential problem underlying the failure to treat AD in humans is the assumption that senile plaques, the most obvious pathological hallmark of the disease, and especially one of the plaque components–Aβ peptide–represent the major driving force of the disease. Here, we challenge this view (see the airbag problem) and review experimental evidence in support of the hypothesis that APP/Aβ belong to a stress/injury-induced protective system of neurons. Hence, we suggest that AD-associated mutations in APP as well as PS1/2 impair an endogenous–protective–function of these proteins, which will in turn result in elevated neuronal vulnerability, diminution of membrane repair following brain injury, and impairments in axonal transport and axonal integrity when cellular stress becomes chronic. In the context of late-onset AD, chronic inflammatory stress to neurons would, with advancing age, override this protective mechanism leading to the same pathological changes as those observed at earlier age in familial AD cases. These impairments would cause synaptic deterioration, neuronal degeneration, and ultimately dementia. In line with this hypothesis and recognized anti-inflammatory properties of Aβ oligomers, senile plaques may represent an ‘aide de camp’ in the neuronal battle against age-associated and inflammation-driven neurodegeneration rather than the cause of neuronal cell death. Finally, understanding the protective mechanism(s) in cognitively healthy elderly people with high plaque and tangle burden, may hold a key for the development of successful treatments for patients already diagnosed with late-onset Alzheimer’s disease.

## Endnote

^a^Estimate of more than 300 published animal model drug trials is based on: Zahs KR, Ashe KH. ‘Too much good news’-are Alzheimer mouse models trying to tell us how to prevent, not cure, Alzheimer’s disease? Trends Neurosci. 2010; 33(8) 381-9.

## Competing interests

Both authors declare that they have no competing interests.

## Search strategy and selection criteria

References for this article were identified through searches of PubMed and using Google search engine with the search terms “Alzheimer”, “amyloid-beta / Aβ”, “amyloid precursor protein / APP”, “neuroprotective”, “beneficial”, “adaptive”, without date restriction. Further articles were also identified through searches of the reference lists of selected articles. Only papers published in English were reviewed. The final reference list was generated on the basis of originality and relevance to the scope of the manuscript.
